# Enhancing agrifood systems with metabolomics: from crop improvement to food quality

**DOI:** 10.3389/fmolb.2026.1766666

**Published:** 2026-02-03

**Authors:** Marina Dantas Corradin, Joanna Lado, Santiago Luzardo, Daniel Vázquez, Facundo Ibáñez

**Affiliations:** 1 Area Agroalimentos INIA, Instituto Nacional de Investigación Agropecuaria, Montevideo, Uruguay; 2 Sistema Vegetal Intensivo, Estación Experimental Salto Grande, Instituto Nacional de Investigación Agropecuaria, Salto, Uruguay; 3 Sistema Ganadero Extensivo, Estación Experimental Tacuarembó, Instituto Nacional de Investigación Agropecuaria, Tacuarembó, Uruguay; 4 Sistema Agrícola-Ganadero, Estación Experimental La Estanzuela, Instituto Nacional de Investigación Agropecuaria, Colonia, Uruguay

**Keywords:** agronomic practices, biomarkers, citrus, meat quality, metabolic pathways, native fruits, olive oil, soybean

## Abstract

Metabolomics has emerged as a prominent tool in agrifood production and agriculture, offering comprehensive insights into the metabolic processes of plants and animals. In Uruguay, where agriculture plays a crucial role in the economy, the application of metabolomics has the potential to significantly enhance the productivity and sustainability of agrifood systems. This review explores its diverse applications, highlighting its role in optimizing crop yield, agrifood quality, and agricultural sustainability. It also emphasizes the transformative impact of metabolomics in advancing agricultural practices and ensuring food security. This review discusses examples of agrifood production, including soybeans, meat, olive oil, citrus fruits, and potential new fruit crops. By providing detailed comprehension into the metabolic processes of plants and animals, metabolomics enables researchers, stakeholders and farmers to make more informed decisions about breeding, cultivation, and production practices. This, in turn, leads to improved crop yields, higher quality agrifood products, and more sustainable agricultural systems.

## 1 Introduction

Agrifood systems refer to the interconnected set of processes, institutions, and infrastructures involved in the production, transformation, distribution, and consumption of food and agricultural products (FAO, 2021). Rather than functioning as simple linear supply chains, these systems are increasingly recognized as dynamic, multi-dimensional socio-ecological networks that influence livelihoods, nutrition, resource use, and innovation. Achieving sustainability in these systems depends on systemic innovation supported by enabling conditions such as regulatory stability, institutional alignment, social acceptance, and investment in research and infrastructure (Herrero et al., 2020). Consequently, agrifood systems are being redefined not only as food production, but as complex platforms for integrated, science-based transformation.

Recent developments in agrifood systems emphasize the integration of digital technologies to support strategic decision-making across increasingly complex value chains. The emergence of Agri-food 4.0 represents a major shift toward intelligent, data-driven frameworks designed to enhance efficiency, traceability, and environmental performance (Belaud et al., 2019). In this context, “omics” science has played a significant role in advancing agrifood research, with particular emphasis on metabolomics. Using advanced analytical instrumentation, high-throughput data acquisition, and machine learning techniques, metabolomics now stands at the forefront of modern food science (Taheri et al., 2024).

The term “Omics” refers to a set of integrative disciplines aimed at analyzing complex interactions within biological systems. It has been widely employed as a holistic framework for investigating biological processes through the application of scientific approaches such as genomics, metabolomics, proteomics, and transcriptomics (Misra et al., 2019; Dai and Shen, 2022). Among these, metabolomics has long been recognized as a relevant approach due to its ability to capture the biochemical phenotype of organisms in response to genetic and environmental factors (Fiehn, 2002). Through the identification and quantification of small-molecule metabolites such as amino acids, organic acids, sugars, and lipids, it provides a direct reflection of physiological and metabolic states. This makes metabolomics especially valuable for investigating responses to stress, disease, nutrition, and environmental changes across diverse biological systems. Within this context, the field of Foodomics has emerged as an integrative framework that brings together omics technologies, with metabolomics serving as a central tool for investigating food composition, nutritional function, and the biological mechanisms through which food components influence health (Cifuentes, 2009; Valdés et al., 2022).

Metabolomics stands out as an essential tool in agrifood systems, as it can be applied across different stages of food production and quality evaluation (Zhang J. et al., 2023). In plant-based systems, it supports cultivar characterization and helps elucidate metabolic processes involved in plant growth, fruit development, and ripening, providing information that can be used to improve product quality and extend shelf life. In food safety contexts, metabolomics is increasingly applied to postharvest studies, authenticity assessment, and traceability, as it enables the identification of metabolites associated with storage-related disorders, the evaluation of pesticide residues, and the detection of adulteration products. In addition, metabolomics is a highly relevant tool for identifying novel bioactive metabolites in food matrices, which may occur naturally in food species or be generated during food processing. Given its versatility and increasing accessibility, metabolomics is particularly valuable in regions where agriculture plays a strategic economic role. Its development and application are especially relevant in countries where crop and livestock production represent core pillars of the economy. This applies to Uruguay, which, with its strong agricultural foundation and prominent position in international food markets, holds significant potential to benefit from scientific advances in this field. The production and export of soybeans, meat, and citrus fruits are fundamental to the economy of the country, whereas olive oil and native fruits are promising new production activities.

This review explores the role of metabolomics within agrifood systems, highlights the strategic importance of agriculture in Uruguay, and analyzes scientific studies that apply metabolomic approaches to relevant agricultural products for Uruguayan economy. The primary objective of presenting this thematic overview is to emphasize the value of metabolomics as a scientific tool for advancing food production and supporting sustainable agricultural practices.

## 2 Role of metabolomics in agrifood systems

Metabolomics is an advanced analytical approach that enables the comprehensive profiling of low molecular weight metabolites in biological systems. In agrifood research, it plays a central role in linking genetic variation and environmental factors to phenotypes, supporting a deeper understanding of plant physiology, animal performance, and food quality (Fiehn, 2002; Tian et al., 2016; Anzano et al., 2022). For example, in soybean, metabolomic studies have shown that variation in amino acids and specialized metabolites, including isoflavones and other polyphenols, reflects the effects of genetic background, developmental stage, and abiotic stress, being associated with phenotypic traits related to seed development and stress responses (Cao et al., 2022). Moreover, in citrus, metabolomic studies indicate that variation in flavonoids, coumarins, terpenoids, sugars, organic acids, and volatile compounds is shaped by differences among species, fruit developmental stage, and environmental conditions, being associated with phenotypic traits linked to fruit maturation, stress responses, and sensory quality (Gaikwad et al., 2025).

Environmental factors are key drivers in metabolomic studies and can be broadly classified as abiotic and biotic. Abiotic factors refer to non-living stressors, including drought, salinity, temperature extremes, solar radiation, and nutrient limitations. In contrast, biotic factors involve living agents such as pathogenic microorganisms, insect pests, competitive weeds, and other biological interactions. By capturing the biochemical responses of organisms to these diverse challenges, metabolomics provides valuable insights for enhancing crop resilience, improving animal health, and optimizing system productivity. Its applications extend throughout the entire agrifood chain, from breeding and cultivation to processing, authentication, and nutritional evaluation.

Metabolomic approaches have become preferred tools for holistic investigations of metabolic processes. These studies can be performed using either untargeted strategies, which aim to maximize metabolite detection across chemically diverse compounds, or targeted strategies, which focus on the quantification of specific, preselected metabolites associated with defined chemical classes or metabolic pathways (Verpoorte et al., 2005). To achieve a comprehensive analysis of the metabolome, high-resolution and high-sensitivity analytical platforms are essential. The most employed techniques include Gas Chromatography-Mass Spectrometry (GC-MS), Liquid Chromatography–Mass Spectrometry (LC-MS), and Nuclear Magnetic Resonance (NMR) Spectroscopy (Verpoorte et al., 2008).

Nowadays, spectroscopic techniques are increasingly used in metabolomics due to their cost-effectiveness and rapid, non-destructive characteristics. In this context, Fourier-transform infrared (FTIR), near-infrared (NIR), and Raman spectroscopy provide fingerprint information reflecting the overall metabolic composition of agricultural products and can be more readily applied as routine analytical tools (Cebi et al., 2023). In contrast, metabolomic studies aiming at a deeper characterization of metabolic pathways and biomarker identification typically rely on more robust spectrometric platforms, such as GC-MS, LC-MS, and NMR, which allow detailed metabolite annotation and quantification (Wu et al., 2022).

One of the major challenges in metabolomic studies is dealing with complex biological matrices, which contain a high diversity of metabolites. In addition, sampling strategies, sample collection, and extraction procedures can significantly influence the resulting metabolic profiles. Furthermore, each analytical platform presents inherent limitations, as LC–MS is affected by ionization efficiency, GC–MS is restricted to volatile or derivatizable metabolites, and NMR spectroscopy has relatively low sensitivity in complex biological samples. However, advances in analytical technologies, including the integration of complementary analytical techniques and the continuous expansion of metabolomic databases, are progressively improving compound identification and enabling a more comprehensive characterization of complex metabolomes.

The interpretation of metabolomic data requires the use of advanced statistical and bioinformatics tools. Commonly applied approaches are unsupervised and supervised learning methods. The former is typically employed for exploratory data analysis and enables the identification of underlying patterns or groupings within the dataset, while the latter is widely used for biomarker discovery, classification, and predictive modeling (Ren et al., 2015). Accordingly, Principal Component Analysis (PCA) and Partial Least Squares-Discriminant Analysis (PLS-DA) are frequently applied as robust multivariate tools. Following these analytical steps, metabolomics helps uncover how metabolic pathways are organized and regulated, highlighting routes involved in natural product biosynthesis relevant to agricultural performance (Dixon et al., 2006). These insights support applications in breeding, agronomy, nutrition, and food innovation.

**FIGURE 1:**
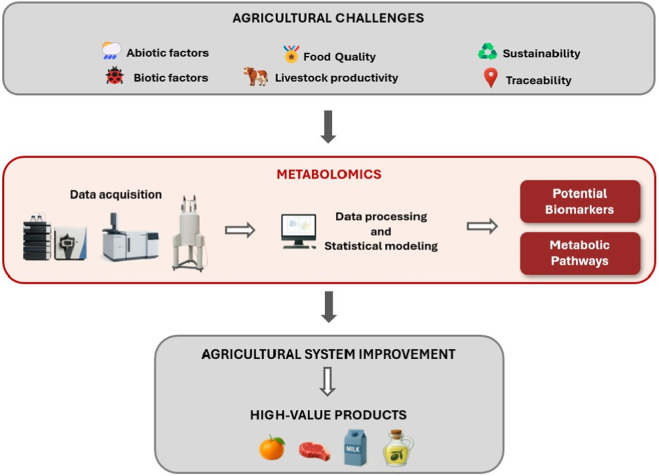
Overview of the role of metabolomics in addressing agrifood system challenges. Metabolomics enables the identification of biochemical responses to a range of agricultural stressors, including abiotic and biotic factors, sustainability concerns, quality, traceability demands, and livestock productivity. Following sample acquisition and analysis using platforms such as LC-MS, GC-MS, or NMR, the data are processed and statistically modeled to identify potential biomarkers and elucidate metabolic pathways. These biomarkers support practical improvements in agricultural systems and contribute to the development of high-value food products, including meat, milk, fruits, and vegetable oils.

Beyond analytical challenges, data analysis in metabolomics also represents an important point of attention. Unsupervised multivariate analyses, such as PCA, may lead to overinterpretation of low-variance components, whereas supervised methods such as OPLS-DA are particularly susceptible to overfitting, especially in datasets with many variables and limited sample sizes. Machine learning has become an integral component of modern metabolomics, serving as a complementary or alternative strategy to classical linear models due to its ability to handle complex and high-dimensional data. Supervised methods, including random forests (RF), support vector machines (SVM), and artificial neural networks (ANNs), are increasingly used to improve predictive accuracy and facilitate the identification of biologically meaningful patterns. These approaches contribute to a more comprehensive understanding of metabolic regulation and system-level behavior (Liebal et al., 2020). To facilitate visualization, Figure 1 provides a schematic overview of the metabolomics workflow and its integration into agrifood systems. The process begins by identifying key challenges in agricultural production, such as abiotic and biotic stress, demands for quality, traceability, sustainability, and livestock productivity. Once samples are analyzed using platforms such as LC–MS, GC-MS, or NMR, the resulting data are processed and modeled. This enables the identification of biomarkers, which are metabolites associated with stress tolerance, growth regulation, or nutritional traits. In addition to biomarker discovery, metabolomics also supports the interpretation of metabolic pathways, helping clarify how metabolic processes respond to agricultural challenges. These findings can then be translated into practical improvements in agriculture, enhancing product quality, and contributing to a more sustainable production chain that delivers higher-value products to consumers.

## 3 Agricultural systems in Uruguay

The agricultural sector, together with livestock, fishing, and associated agro-industrial activities, plays a central role in Uruguay’s economy, contributing substantially to the country’s gross domestic product (GDP). In 2023, agrifood exports made up 80% of Uruguay’s total goods exported, underscoring the country’s reliance on agricultural trade (Uruguay XXI, 2024a). The main agricultural exports include soybeans, rice, wheat, citrus fruits, and olive oil, whereas livestock production remains the leading activity within Uruguay’s agrifood sector and a cornerstone of its export economy (Uruguay XXI, 2024b). Uruguayan meat is exported to more than 100 countries, with China, the United States, and the European Union among the most prominent destinations.

The National Institute of Agricultural Research (INIA) in Uruguay plays a central role in generating, adapting, and transferring technologies and knowledge that address the specific needs of the national agrifood sector (INIA, 2024). These institutional initiatives emphasize Uruguay’s growing capacity to incorporate advanced analytical tools, such as metabolomics, into agricultural research and innovation. Expanding the application of metabolomics across both crop and livestock systems could improve breeding strategies, enhance product differentiation, and optimize nutritional outcomes.

Currently, research groups in Uruguay are initiating metabolomic approaches to a range of nationally relevant products, including beef, soybean, olive oil and mandarins. Preliminary studies aimed to understand how factors like diet, genotype, and cultivation conditions influence nutritional composition, quality traits, and consumer acceptance. Examples include the impact of diet on beef quality (Luzardo et al., 2021), variability in pecan nutritional composition (Ferrari et al., 2022), sensory-driven selection of mandarin cultivars; (Migues et al., 2021; Migues et al., 2022), the effects of irrigation on olive oil quality (Conde-Innamorato et al., 2022), and support food safety of fruits and vegetables (Pereira et al., 2021).

Although metabolomics has been applied in some national studies, its full potential remains underexplored. Expanding its use could enhance agricultural productivity through the discovery of new biomarkers, optimization of protocols, and integration of advanced data analysis techniques. Investing in this field can support more precise decision-making, and drive innovations tailored to local crops, breeds, and environmental conditions. As the global demand for sustainable, high-quality agrifood products continues to rise, metabolomics represents a relevant path to enhance competitiveness within the agricultural sector.

## 4 Metabolomic applications in strategic agrifood products of Uruguay

Building on the strategic overview of Uruguay’s agricultural systems, this section reviews some of the recent international studies applying metabolomics to agrifood products of national relevance: soybean, meat, olive oil, citrus fruits, and native plant species. Together, these studies illustrate how metabolomics has been successfully applied worldwide to address similar production systems and commodities, reinforcing opportunities for scientific advancement at the national level.

The references used in this review were retrieved from the Scopus, PubMed and Google Scholar databases. Studies were identified using keywords such as metabolomics, metabolomic studies, and related variations, combined with terms referring to agrifood products of interest including soybean, meat, olive oil and citrus fruits. For the native plant species, fruit-bearing species of interest were considered: *Butia odorata*, *Eugenia uniflora*, *Feijoa sellowian*a, and *Psidium cattleianum*. The search results were filtered by publication year, and studies published more than 8 years prior were excluded. Following this, studies were screened based on analytical methodology, and only those employing NMR, LC-MS, or GC-MS approaches were included. The selected studies were then examined in greater detail, and those focusing on species or challenges not directly relevant to the Uruguayan agrifood context were excluded. Finally, for each agrifood product, the studies were grouped into thematic application domains, as summarized in Table 1. By synthesizing international evidence, this section provides a background that supports the expansion of metabolomics research within Uruguay’s agrifood sector.

**TABLE 1:** Thematic categories of metabolomics applications in Uruguay’s strategic agrifood products, including soybeans, meat, olive oil, citrus fruits, and native species.

Product	Areas of metabolomic applications
Soybeans	Nutritional value and quality Strategies for crop improvement Traceability and origin classification
Meat	Quality and composition Animal growth and performance Diet and product traits
Olive oil	Adulteration detection and origin classification Cultivation and agronomic practices Production and processing effects
Citrus fruits	Species or varietal identification/characterization Fruit development and composition Quality traits and variation Stress response and defense Product adulteration
Native fruits	Fruit development and ripening Bioactive compounds and applications Compositional traits and variability Plant defense compounds

### 4.1 Soybean metabolomics

Soybean is not only a primary global source of plant-based protein but also a crop of strategic importance for sustainable agriculture, contributing to crop rotation systems and international trade. Beyond its content of protein, oil, fatty acids, and sugars, soybean tissues, including seeds, leaves, and roots, contain a wide array of bioactive metabolites, which contribute to plant development and environmental resilience, offering strategic targets for modern breeding and smart agriculture (Mani et al., 2024). In response to increasing demand for nutritional quality and climate-resilient cultivars, recent research has employed metabolomics to investigate key physiological and biochemical processes in soybean. Table 2 presents selected studies that illustrate these advances, summarizing their main findings, analytical approaches, and categorizing them into three thematic areas of research:

- Nutritional value and quality: Explore how environmental conditions, genetic factors, and developmental stages influence seed composition, particularly with respect to amino acids, flavonoids, and lipid profiles.
- Agronomic strategies for crop improvement: Discuss metabolic pathways involved in stress tolerance, root symbiosis, and hormone signaling.
- Traceability and origin classification: Investigate biomarkers used to differentiate soybean varieties, production systems, and geographic origins.

**TABLE 2:** Summary of recent studies on soybean metabolomics, including analytical platforms and major findings across nutritional, agronomic, and traceability applications.

Investigation fields	Main findings	Technique	References
Nutritional value and quality	Potassium availability alters soybean metabolism and reveals L-asparagine as a biomarker.	LC-MS	Cotrim et al. (2023)
	Soybean mutant shows higher seed protein and metabolic changes.	LC-MS	Islam et al. (2023)
	Soybean node position affects the seed metabolite content during development.	LC-MS	Takpah et al. (2023)
	Soybean germination enhances flavonoid metabolite content and activates new biosynthetic pathways.	LC–MS	Bi et al. (2022a)
	Heat stress during transport alters soybean metabolism and reduces nutritional quality.	LC-MS	Zhu et al. (2022a)
	Genes associated with seed development control protein and oil content in soybeans.	LC-MS	Xu et al. (2022)
	Hydrogen treatment boosts isoflavone aglycones and mitigates UV-B stress in germinated soybeans.	LC-MS	Xu et al. (2024)
	Higher growth temperatures influence wild soybean metabolite profiles, especially amino acid composition.	GC-MS	Bao et al. (2023)
	Exploration of new bioactive metabolites in wild soybeans	LC-MS	Nguyen et al. (2024)
	Silver treatments caused minor changes in soybean metabolism.	NMR	Quintela et al. (2024)
Strategies for crop improvement	Exogenous nitrogen inhibits nodule growth and nitrogen fixation by altering root nodule metabolism.	LC-MS	Lyu et al. (2022b)
	Nitrogen and phosphorus deficiencies trigger distinct molecular responses in soybean roots	LC-MS	Nezamivand-Chegini et al. (2023)
	Soybean shows enhanced flavonoid biosynthesis linked to key regulatory genes under salt stress.	LC-MS	Wang et al. (2024d)
	Key genes and metabolic pathways regulate soybean salt tolerance, offering targets for crop improvement.	LC-MS	Fu et al. (2024)
	Soybean resistance to anthracnose involves hormonal signaling, defense genes, and terpenoid metabolism.	LC-MS	Zhu et al. (2022b)
	Humic materials promote nodule formation in soybeans by regulating hormones.	LC-MS	Zhang et al. (2023b)
	Exogenous melatonin alleviates alkaline stress in soybeans through metabolic and gene regulation.	LC-MS	Duan et al. (2024)
	Combined water deficit and heat stress lead to distinct metabolic responses and reveal specific biomarkers in soybeans.	LC-MS and GC-MS	Vital et al. (2022)
	Crop resilience guided by metabolic markers.	LC-MS and GC-MS	Razzaq et al. (2022)
	Chlorogenic acid and early metabolic responses enhance soybean resistance to root rot caused by *Fusarium tricinctum*.	GC-MS	Zhang et al. (2025b)
	Root metabolic and genetic adaptations to low-phosphorus stress differentiate wild soybeans.	GC-MS	Li et al. (2022b)
Traceability and origin classification	The quality characteristics of black soybeans from different geographical origins.	LC-MS	He et al. (2023)
	Wild and cultivated soybeans distinguished by metabolomics.	LC-MS	Tareq et al. (2023)
	Integrated metabolomics and transcriptomics enable accurate soybean traceability by geographic origin.	LC–MS	Wang et al. (2024a)
	Flavonoid profiles in soybean leaves vary with growth stage and environment, as revealed by targeted metabolomics and machine learning.	LC-MS	Rha et al. (2023)

The reviewed soybean studies report that metabolites such as amino acids, flavonoids, and isoflavones are highly associated with environmental conditions, developmental stage, and post-harvest handling. In addition, flavonoid and terpenoid pathways are associated with salt tolerance, nutrient deficiency, pathogen resistance, and combined stress scenarios. Metabolomic fingerprints also show consistent success in discriminating against soybean varieties and geographic origins. These findings indicate that soybean metabolic profiles reflect dynamic interactions between genotype, growth conditions, and stress exposure. However, variations observed across studies are mostly attributable to differences in genotypes, growth stage and environmental conditions.

### 4.2 Meat metabolomics

Metabolomic approaches have been applied to investigate key aspects such as physiological responses, the evaluation of nutritional strategies, and the characterization of meat quality attributes. Given the central contribution of meat production to Uruguay’s agrifood economy, the local utilization of metabolomics would be highly significant for maintaining and enhancing competitiveness. Table 3 presents an overview of recent studies that employed metabolomics in livestock systems, aiming to identify biomarkers associated with genetic background, nutrient metabolism, and commercial meat quality traits. These studies are organized into three thematic categories:

- Meat quality and composition: Investigate beef tenderness, meat ageing/spoilage, and how metabolic compounds influence texture, flavor, and shelf-life.
- Animal growth and performance: Identify metabolites associated with feed efficiency, energy metabolism, and muscle growth.
- Diet and product traits: Evaluate how different diets or supplements affect the metabolic status and meat traits.

**TABLE 3:** Overview of recent metabolomics studies in meat production, highlighting analytical techniques and key findings related to quality traits, animal growth, and dietary effects.

Investigation fields	Main findings	Technique	References
Meat quality and composition	Biomarkers for fat color identified by distinguishing metabolites in white and yellow fat.	LC-MS	Tian et al. (2023)
	Early castration enhances beef marbling by altering liver metabolism and gene expression.	LC-MS	Sun et al. (2022)
	Comparing pasture and grain finished beef using metabolomics.	LC-MS	Evans et al. (2024)
	Spoilage-related metabolic changes and biomarkers identified during chilled beef storage using metabolomics.	LC-MS	Liu et al. (2025a)
	Metabolic changes during dry-aging identified by profiling time-dependent metabolites.	LC-MS	Sun et al. (2024)
	Metabolic and quality differences were identified across aging methods, revealing distinct compound profiles and effects on beef tenderness, color, and stability.	LC-MS	Liu et al. (2025c)
	Tenderness-related metabolic fingerprints identified during postmortem aging of beef.	LC-MS and GC-MS	King et al. (2019)
	Breed-specific beef quality is identified by comparing physicochemical and metabolic profiles.	NMR	Phoemchalard et al. (2022)
Animal growth and performance	Biomarkers identified in steers with divergent growth performance using metabolomics.	LC-MS	Artegoitia et al. (2022)
	Metabolites linked to carcass traits identified by integrating metabolomics, genomics, and phenotypes.	NMR	Li et al. (2022a)
	Growth rate and finishing system influence beef muscle metabolism, especially energy, protein, and lipid pathways.	NMR	Gómez et al. (2022)
	Growth-related metabolic signatures identified in grazing cattle.	NMR	Imaz et al. (2022)
Diet and product traits	Long-term metabolic effects of prenatal nutrition identified through integrated metabolome–microbiome analysis in Nelore bulls.	LC-MS	Polizel et al. (2025)
	Metabolic and reproductive benefits by creep feeding in Nelore heifers.	LC-MS	Catussi et al. (2024)
	The effects of high dietary energy density on the metabolism of transition Angus cows revealed by metabolomics	LC–MS	Chen et al. (2022a)
	Prenatal supplementation in beef cattle and its effects on amino acid metabolism	LC-MS	Schalch Junior et al. (2022)
	Beef and postprandial metabolic differences identified by comparing grass-fed and conventional feeding systems.	LC-MS	Spears et al. (2024)
	Effects on meat quality of 3-nitrooxypropanol in feedlot beef cattle diets	NMR	Pedrini et al. (2024)

The literature examined identifies metabolic variation among meat samples associated with aging methods, divergent growth rates, storage conditions, breed, and postmortem quality traits such as tenderness. In nutritional intervention studies, metabolic responses often extend beyond immediate dietary effects, influencing meat quality attributes and physiological performance. The variability reported across studies can be attributed to differences in production systems and experimental design.

### 4.3 Olive oil metabolomics

Olive oil production has gained importance in Uruguay in recent years, led by increasing interest in high-value, health-oriented agrifood products. Recent studies have employed metabolomics to characterize oil composition, monitor quality, and detect adulteration, especially for extra virgin olive oil (EVOO) due to its high commercial value. This approach also helps evaluate how factors such as cultivar, irrigation, and processing methods influence the chemical profile of olive oil. Table 4 summarizes recent studies focused on identifying biomarkers linked to origin, authenticity, production conditions, and functional properties. The research is grouped into four categories:

- Adulteration detection and origin classification: Identify biomarkers that distinguish extra virgin olive oil from adulterated products and enable geographic or varietal classification, supporting traceability and consumer confidence.
- Cultivation and agronomic practices: Investigate how factors like irrigation, drought, and pathogen resistance alter metabolic pathways in olive tissues.
- Production and processing effects: Investigate how to harvest stage, extraction method, and postharvest treatments significantly influence the chemical profile of olive oil.

**TABLE 4:** Overview of recent olive oil metabolomics studies with a focus on authenticity, traceability, agronomic practices, and processing methods.

Investigation topics	Main findings	Technique	References
Adulteration detection and origin classification	Phenolic and sterolic fingerprints accurately differentiate EVOO by cultivar and geographical origin.	LC-MS	Ghisoni et al. (2019)
	Sterol and phenolic profiling enable high-accuracy authentication of EVOO.	LC-MS	Senizza et al. (2023)
	Commercial olive-based supplements show high compositional variability	LC-MS	Garcia-Aloy et al. (2020)
	Untargeted metabolomic profiling detects camellia oil adulteration in EVOO with discriminatory biomarkers.	LC-MS	Dou et al. (2025)
	Advanced metabolomic analysis enables EVOO adulteration detection with high sensitivity and expanded metabolite coverage.	LC-MS	Drakopoulou et al. (2024)
	Combined phenolic and sensory profiling distinguishes geographically certified and commercial EVOOs.	LC-MS and GC-MS	Ros et al. (2019)
	Metabolite profiling enables rapid and comprehensive classification of olive oil cultivars.	NMR	Tang et al. (2022)
	Comparative metabolomic analysis improves marker discovery and strengthens data validation in oil studies.	NMR	Schripsema (2019)
	Metabolite fingerprinting combined with classification models accurately verifies the geographical origin of virgin olive oils.	NMR	Alonso-Salces et al. (2025)
Cultivation and agronomic practices	Metabolomic profiling across olive organs reveals key markers associated with resistance to *Verticillium wilt*.	LC-MS	Serrano-García et al. (2024)
	Olive mill wastewater mitigates drought stress in wheat by enhancing metabolite profiles and antioxidant capacity.	LC-MS	Hamoud et al. (2025)
	Olive leaf polyphenol profiles vary by cultivar and season, impacting antioxidant potential.	LC-MS	Difonzo et al. (2022)
	Metabolomics reveal cultivar-specific phenolic and lipid responses in olive leaves and stems.	LC-MS and GC-MS	Parri et al. (2024)
Production and processing effects	Metabolomic profiling reveals olive oil by-products as rich sources of phenolics and terpenes for valorization.	LC-MS	López-Salas et al. (2024)
	Olive by-products from different oil presses exhibit distinct metabolite profiles, particularly in phenolics and fatty acids.	LC-MS and GC-MS	Fayek et al. (2024)
	Pickling process impacts the olive metabolome and sensory properties.	LC-MS and GC-MS	Fayek et al. (2021)
	Multi-omics analysis defines optimal olive harvest strategies based on metabolite and gene expression profiles.	LC-MS and GC-MS	Rao et al. (2021)
	Integrated metabolite profiling reveals antioxidant-rich compounds in olive by-products.	LC-MS and NMR	Zahran et al. (2025)
	Ultrasound-assisted extraction enhances bioactive compounds in EVOO across ripening stages.	NMR	Del Coco et al. (2021)

Metabolomics studies of olive oil show strong agreement that oil production processes and environmental conditions shape olive metabolic profiles. In the context of adulteration detection and origin classification, studies demonstrate that phenolic fingerprints are robust biomarkers for geographical origin verification and adulterant detection. Furthermore, studies reveal metabolic signatures associated with stress responses, seasonal variation, and antioxidant capacity. Differences among studies are mainly explained by variation in sampling design and processing techniques. Overall, these studies highlight the versatility of metabolomics for addressing authenticity, sustainability, and value creation across the olive oil value chain.

### 4.4 Citrus fruit metabolomics

Citrus fruits are a main component of Uruguay’s fruit production and industry, with considerable importance for domestic consumption, industry and international trade. Their commercial value depends on traits such as flavor, sweetness, acidity, nutritional content, and postharvest stability. Metabolomics has significantly contributed to the understanding of citrus biology by elucidating mechanisms involved in plant and fruit development, species and varietal differentiation, quality attributes, product adulteration and responses to biotic and abiotic stress. In this regard, one of the most studied challenges in citrus production is Huanglongbing (HLB), also known as citrus greening, a devastating disease associated primarily with the bacterium *Candidatus Liberibacter asiaticus* (CLas). Table 5 summarizes recent studies that apply metabolomic approaches to citrus fruit and derived products such as juices. These investigations focus on metabolite profiling related to fruit quality, species/varietal identification, physiological shifts, potential product adulteration and stress adaptation, and are grouped into five categories:

- Species or varietal identification/characterization: Identification of candidate metabolites or biomarkers for separation of citrus species and varieties.
- Fruit development and composition: Explore changes in metabolites during citrus growth and ripening, offering insights into fruit physiology and nutritional value.
- Quality traits and variation: Identifying compounds linked to flavor, aroma, and nutritional parameters.
- Stress response and defense: Analyze defense-related metabolites and pathways activated by pathogens or environmental stressors.
- Product adulteration: Identify key adulterants or diverse products in citrus juices.

**TABLE 5:** Overview of recent citrus fruit metabolomics studies addressing development, quality traits, and stress responses.

Investigation topics	Main findings	Technique	References
Species/varietal identification and characterization	Several key secondary metabolites such as polymethoxyflavones, furanocoumarins and volatiles were identified to be potential biomarkers for separation of citrus species.	LC-MS	Goh et al. (2022)
	Differential accumulation of flavonoids and coumarins among citrus species reveals metabolic variation relevant to bioactivity and supports breeding strategies aimed at enriching beneficial flavonoids and minimizing the potential risks associated with coumarins.	LC-MS	Liang et al. (2024)
	Integrated metabolomic and genomic analyses revealed interspecific variation in bioactive phenylpropanoids and enabled species-level discrimination in Citrus.	LC-MS	Wang et al. (2024c)
Fruit and plant development and composition	Citrus exocarp concentrates key bioactives, with distinct metabolomic profiles across fruit tissues.	LC-MS	Dadwal et al. (2022)
	Metabolite profiles in immature citrus vary by cultivar and fruit size, revealing dynamic changes in early fruit development.	LC-MS	Deschamps et al. (2024)
	Seven new natural sweeteners and sweetness were identified with potential use for breeding or industry. The proposed screening strategy could boost the identification of key natural taste modulators.	LC-MS	Wang et al. (2022a)
	Limonoid composition varies across pummelo tissues, with seeds showing the highest diversity and abundance.	LC-MS	Liu et al. (2021b)
	Peel roughness in lemons is associated with altered hormonal signaling and reduced terpenoid biosynthesis.	LC-MS	Liu et al. (2021a)
	Citrus fruit development is marked by distinct metabolic shifts, including a decline in phenolics and an increase in carotenoids.	LC-MS and GC-MS	Kim et al. (2022)
	A novel log-ratio-based approach (reducing inter-study bias) to compare metabolite profiles between fruits and leaves across major citrus groups.	LC-MS	Traband et al. (2025)
Quality traits and their variation	Rootstocks modulate key metabolic pathways and specialized metabolites affecting fruit quality in late-maturing hybrid mandarins.	LC-MS	Wang et al. (2024b)
	Integrated metabolome–transcriptome analysis reveals key regulators of fruit quality in mandarin–orange hybrids.	LC-MS	Bi et al. (2022b)
	Higher levels of flavonoids, amino acids and derivatives, terpenoids, and alkaloids differences are associated with peel roughness defect in Orah mandarins	LC-MS	Liu et al. (2025b)
	Rootstocks modulate key metabolic pathways in HLB-affected orange juice.	LC-MS	Liu et al. (2023)
	Naringin and neohesperidin are the primary contributors to bitterness in pummelo.	LC-MS	Xu et al. (2025)
	Low-cost benchtop analysis predicts sugar and acid levels and supports consumer preference modeling with simplified chemometrics.	NMR	Migues et al. (2022)
	Characterization and comparison of the morphological and biochemical properties of the late-season varieties juices.	NMR	Maciá-Vázquez et al. (2023)
	Combined ^1^H-NMR and HPLC ensured precise *Citrus* juice authentication based on chemical markers to improve juice differentiation.	NMR and HPLC	Jungen et al. (2025)
	Spectral profiling distinguishes mandarin cultivars and predicts consumer preference based on sugar–acid balance.	NMR	Migues et al. (2021)
Stress response and induced defenses	Exogenous naringin delays citrus decay by activating flavonoid biosynthesis and enhancing antioxidant defenses.	LC-MS	Zeng et al. (2022)
	Early metabolic responses to CLas infection differ by citrus tolerance, revealing biomarkers for HLB resistance.	LC-MS	Li et al. (2024)
	HLB-tolerant mandarins sustain growth via auxin, cytokinin, and purine metabolism rather than salicylic acid defense.	LC-MS	Suh et al. (2021)
	Early CLas infection alters sugar, amino acid, and fatty acid metabolism without visible leaf symptoms.	LC-MS	Chen et al. (2022b)
Stress Response and Defense	HLB disrupts peel pigmentation in mandarin by altering phenylpropanoid and flavonoid metabolism.	LC-MS	Wang et al. (2020)
	Cold tolerance in *Citrus* is linked to high accumulation of sphingosine, chlorogenic acid, and other stress-related metabolites.	LC-MS	Xiao et al. (2024)
Functional Compounds and Applications	Early asymptomatic HLB detection in citrus is achievable with high accuracy using metabolomics and machine learning.	LC-MS	Wang et al. (2022b)
	*Penicillium digitatum* infection alters citrus pulp metabolism and activates hormone-mediated defense pathways.	GC-MS	Tang et al. (2018)
	Postharvest treatments in Satsuma mandarin prevent fungal decay by increasing levels of total phenolics, flavonoids and other secondary metabolites.	LC-MS	Duan et al. (2023)
	HLB infection alters the citrus root metabolome and microbiome in a variety-specific manner.	NMR	Padhi et al. (2019)
Product adulteration	Untargeted screening coupled with machine learning models can be a powerful tool to facilitate detection of lemon juice adulteration.	LC-MS	Lyu et al. (2022a)
	Untargeted metabolomics is useful to identify the differences between orange juices from concentrated or not. A total of 91 and 42 potential markers were defined in positive/negative mode.	LC-MS	Xu et al. (2020)
	Volatile fingerprinting accurately predicts lemon juice quality parameters and discriminates varieties, enabling authenticity assessment.	GC-MS	Giménez-Campillo et al. (2025)

The studies presented in Table 5 show that metabolites, including flavonoids, coumarins, and volatile compounds, act as robust biochemical markers enabling reliable discrimination among citrus species. In terms of quality traits, metabolomics studies identify biomarkers as key determinants of sensory attributes such as bitterness, sweetness, aroma, and peel texture. In addition, studies focused on stress responses present strong convergence by revealing asymptomatic metabolic alterations associated with pathogen infection and abiotic stress tolerance, highlighting the potential of metabolomics for early stress detection and resistance screening. In product authentication and adulteration detection, metabolomic fingerprinting demonstrates high accuracy and robustness in differentiating juice origin and adulteration.

### 4.5 Native fruits metabolomics

Some native fruits in Uruguay, such as butiá (*Butia odorata*), pitanga (*Eugenia uniflora*), guayabo (*Feijoa sellowiana*), and arazá (*Psidium cattleianum*), have attracted increasing interest due to their antioxidant properties, distinctive phytochemical profiles, and potential as emerging fruit crops. However, these species remain largely underexplored, underlining their value as promising targets for scientific research and sustainable innovation.

Metabolomics is a suitable method for studying these native fruits. It helps reveal biochemical pathways related to their development, chemical composition, defense responses to environmental stresses, and functional properties. This knowledge supports their valorization, fosters bioeconomic opportunities, and promotes the sustainable use of native biodiversity linked to local food traditions.

Table 6 presents a selection of recent studies focused on these fruits, summarizing their main findings, analytical platforms and grouping them into four thematic categories:

- Fruit development and ripening: Explore the biochemical and hormonal changes involved in fruit maturation and the tissue-specific accumulation of metabolites.
- Bioactive compounds and applications: Characterize phytochemicals with antioxidant, antitumor, or antifungal properties, highlighting their potential use in food, pharmaceutical, and cosmetic applications.
- Compositional traits and variability: Investigate chemical diversity across species, cultivars, geographic origins, and postharvest conditions.
- Plant defensive compounds: Characterize metabolites involved in antifungal and antimicrobial defense mechanisms.

**TABLE 6:** Overview of recent metabolomics studies on native Uruguay’s fruits, focusing on development, bioactive compounds, and variability.

Investigation topics	Main findings	Technique	References
Fruit development and ripening	Key metabolic pathways during guava ripening are modulated by ethylene and abscisic acid.	LC-MS	Monribot-Villanueva et al. (2022)
	Ripening stages in feijoa fruits are distinguished by changes in volatile compounds.	GC-MS	Song et al. (2023)
	Volatilome study of feijoa fruit revealed metabolic pathways involved in aroma biosynthesis.	GC-MS	Baena-Pedroza et al. (2020)
	Metabolite profiles variation of *Eugenia uniflora* fruits during ripening.	GC-MS	Pascoal et al. (2025)
Bioactive compounds and applications	The production of high-value bioactive compounds by *Feijoa*.	LC-MS	Raikar et al. (2023)
	Antioxidant and antitumor properties of *Butia odorata* fruit are linked to its phenolic profile.	LC-MS	Boeing et al. (2020)
	Extracts from *Psidium cattleianum* fruits and leaves modulate cation channel receptors.	LC-MS	Zhang et al. (2025a)
	Evaluation of polyphenols and antioxidants in Feijoa flowers.	LC-MS	Montoro et al. (2020)
Compositional traits and their variability	Metabolite profiles distinguish *Butia* species and growing locations.	LC-MS	Hoffmann et al. (2017a)
	Processing and storage affect the content of bioactive compounds in *Butia odorata* products.	LC-MS	Hoffmann et al. (2017b)
	Volatile and metabolic profiles of pitanga fruits vary by color and ripening stage, revealing compositional diversity.	GC-MS	Pascoal et al. (2025)
Plant defense compounds	Antifungal activity of *Eugenia uniflora* leaf extracts is associated with major polyphenols such as myricitrin and ellagic acid.	LC-MS	Tenório et al. (2024)
	Bioactivity-guided metabolite profiling of Feijoa reveals a potent antifungal inhibitor.	GC-MS	Mokhtari et al. (2018)

Despite the relatively limited number of investigations compared with major crops, some studies have addressed at least one native fruit species. Metabolomic approaches enable robust discrimination of species, growing locations, fruit development and ripening stages based on volatile and non-volatile metabolic fingerprints. Moreover, studies demonstrate that both genetic background and postharvest handling significantly affect metabolite profiles, influencing nutritional quality and functional properties. These observations indicate that native species including butiá, pitanga, guayabo, and arazá remain insufficiently explored by metabolomic approaches, representing an important opportunity for future scientific research and commercial development in Uruguay.

## 5 Conclusion and perspectives

Metabolomics has become an essential approach in agrifood research, increasingly applied to improve agricultural practices and ensure food security. By providing integrated insights into plant- and animal-based systems, it enables the identification of biomarkers associated with product quality, nutritional attributes, and environmental adaptation. These advances contribute to more informed decision-making in breeding, cultivation, livestock management, and postharvest processes, directly influencing productivity, product quality, and the sustainability of agrifood systems.

As previously mentioned, agrifood production plays a predominant position in Uruguay’s economy, led by commodities such as soybeans, meat, and citrus fruits, along with initial olive oil industry, and potential new crops derived from native fruits. In this review, we examined recent international studies that investigated these agrifood products using metabolomic approaches. Among the most frequently studied themes are: (i) metabolic features related to composition, nutritional value, quality, and functional attributes, and (ii) production and environmental responses, including cultivation practices, processing effects, traceability, and authenticity.

Within this context, metabolomics emerges as an effective strategy for developing high-quality agrifood products. Through the application of this approach and the use of appropriate analytical platforms, continued progress is expected, driving innovation in the development of resilient and sustainable agrifood systems.

## References

[B1] Alonso-Salces R. M. Viacava G. E. Tres A. Vichi S. Valli E. Bendini A. (2025). Stepwise strategy based on untargeted metabolomic 1H NMR fingerprinting and pattern recognition for the geographical authentication of virgin olive oils. Food Control 173, 111216. 10.1016/j.foodcont.2025.111216

[B2] Anzano A. Bonanomi G. Mazzoleni S. Lanzotti V. (2022). Plant metabolomics in biotic and abiotic stress: a critical overview. Phytochem. Rev. 21, 503–524. 10.1007/s11101-021-09786-w

[B3] Artegoitia V. M. Newman J. W. Foote A. P. Shackelford S. D. King D. A. Wheeler T. L. (2022). Non-invasive metabolomics biomarkers of production efficiency and beef carcass quality traits. Sci. Rep. 12, 231. 10.1038/s41598-021-04049-2 34997076 PMC8742028

[B4] Baena-Pedroza A. Londoño-Giraldo L. M. Taborda-Ocampo G. (2020). Volatilome study of the feijoa fruit [Acca sellowiana (O. Berg) Burret.] with headspace solid phase microextraction and gas chromatography coupled with mass spectrometry. Food Chem. 328. 10.1016/j.foodchem.2020.127109 32454261

[B5] Bao G. Mu L. Wang Y. (2023). Effect of different accumulative temperate zones in Heilongjiang on Glycine soja metabolites as analyzed by non-target metabolomics. Molecules 28, 3296. 10.3390/molecules28083296 37110529 PMC10143369

[B6] Belaud J. P. Prioux N. Vialle C. Sablayrolles C. (2019). Big data for agri-food 4.0: application to sustainability management for by-products supply chain. Comput. Ind. 111, 41–50. 10.1016/j.compind.2019.06.006

[B7] Bi W. Zhao G. Zhou Y. Xia X. Wang J. Wang G. (2022a). Metabonomics analysis of flavonoids in seeds and sprouts of two Chinese soybean cultivars. Sci. Rep. 12, 5541. 10.1038/s41598-022-09408-1 35365712 PMC8975843

[B8] Bi X. Liao L. Deng L. Jin Z. Huang Z. Sun G. (2022b). Combined transcriptome and metabolome analyses reveal candidate genes involved in tangor (citrus reticulata × citrus sinensis) fruit development and quality formation. Int. J. Mol. Sci. 23, 5457. 10.3390/ijms23105457 35628266 PMC9141862

[B9] Boeing J. S. Barizão É. O. Rotta E. M. Volpato H. Nakamura C. V. Maldaner L. (2020). Phenolic compounds from Butia odorata (Barb. Rodr.) noblick fruit and its antioxidant and antitumor activities. Food Anal. Methods 13, 61–68. 10.1007/s12161-019-01515-6

[B10] Cao P. Zhao Y. Wu F. Xin D. Liu C. Wu X. (2022). Multi-omics techniques for soybean molecular breeding. Int. J. Mol. Sci. 23, 4994. 10.3390/ijms23094994 35563386 PMC9099442

[B11] Catussi B. L. C. Ferreira J. R. Lo Turco E. G. Morgulis S. C. F. Baruselli P. S. (2024). Metabolic imprinting in beef calves supplemented with creep feeding on performance, reproductive efficiency and metabolome profile. Sci. Rep. 14, 9702. 10.1038/s41598-024-60216-1 38678099 PMC11055875

[B12] Cebi N. Bekiroglu H. Erarslan A. (2023). Nondestructive metabolomic fingerprinting: FTIR, NIR and raman spectroscopy in food screening. Molecules 28, 7933. 10.3390/molecules28237933 38067662 PMC10707828

[B13] Chen H. Wang C. Huasai S. Chen A. (2022a). Metabolomics reveals the effects of high dietary energy density on the metabolism of transition angus cows. Animals 12, 1147. 10.3390/ani12091147 35565573 PMC9105006

[B14] Chen Q. Min A. Luo S. He J. Wu R. Lin X. (2022b). Metabolomic analysis revealed distinct physiological responses of leaves and roots to huanglongbing in a citrus rootstock. Int. J. Mol. Sci. 23, 9242. 10.3390/ijms23169242 36012507 PMC9409271

[B15] Cifuentes A. (2009). Food analysis and foodomics. J. Chromatogr. A 1216, 7109. 10.1016/j.chroma.2009.09.018 19765718

[B16] Conde-Innamorato P. García C. Villamil J. J. Ibáñez F. Zoppolo R. Arias-Sibillotte M. (2022). The impact of irrigation on olive fruit yield and oil quality in a humid climate. Agronomy 12. 10.3390/agronomy12020313

[B17] Cotrim G. dos S. Silva D. M. Graça J. P. Oliveira Junior A. Castro C. (2023). Glycine max (L.) Merr. (soybean) metabolome responses to potassium availability. Phytochemistry 205, 113472. 10.1016/j.phytochem.2022.113472 36270412

[B18] Dadwal V. Joshi R. Gupta M. (2022). A comparative metabolomic investigation in fruit sections of citrus medica L. and citrus maxima L. detecting potential bioactive metabolites using UHPLC-QTOF-IMS. Food Res. Int. 157, 111486. 10.1016/j.foodres.2022.111486 35761710

[B19] Dai X. Shen L. (2022). Advances and trends in omics technology development. Front. Med. (Lausanne) 9, 911861. 10.3389/fmed.2022.911861 35860739 PMC9289742

[B20] Del Coco L. Girelli C. R. Angilè F. Mascio I. Montemurro C. Distaso E. (2021). NMR-based metabolomic study of apulian coratina extra virgin olive oil extracted with a combined ultrasound and thermal conditioning process in an industrial setting. Food Chem. 345, 128778. 10.1016/j.foodchem.2020.128778 33310250

[B21] Deschamps E. Durand-Hulak M. Castagnos D. Hubert-Roux M. Schmitz I. Froelicher Y. (2024). Metabolite variations during the first weeks of growth of immature citrus sinensis and citrus reticulata by untargeted liquid chromatography–mass spectrometry/mass spectrometry metabolomics. Molecules 29, 3718. 10.3390/molecules29163718 39202798 PMC11357260

[B22] Difonzo G. Crescenzi M. A. Piacente S. Altamura G. Caponio F. Montoro P. (2022). Metabolomics approach to characterize green olive leaf extracts classified based on variety and season. Plants 11, 3321. 10.3390/plants11233321 36501360 PMC9735528

[B23] Dixon R. A. Gang D. R. Charlton A. J. Fiehn O. Kuiper H. A. Reynolds T. L. (2006). Applications of metabolomics in agriculture. J. Agric. Food Chem. 54, 8984–8994. 10.1021/jf061218t 17117782

[B24] Dou X. N´Diaye K. Harkaoui S. E. Willenberg I. Ma F. Zhang L. (2025). Authentication of virgin olive oil based on untargeted metabolomics and chemical markers. Eur. J. Lipid Sci. Technol. 127, e202400126. 10.1002/ejlt.202400126

[B25] Drakopoulou S. K. Kritikou A. S. Baessmann C. Thomaidis N. S. (2024). Untargeted 4D-metabolomics using trapped ion mobility combined with LC-HRMS in extra virgin olive oil adulteration study with lower-quality olive oils. Food Chem. 434, 137410. 10.1016/j.foodchem.2023.137410 37708573

[B26] Duan B. Tan X. Long J. Ouyang Q. Zhang Y. Tao N. (2023). Integrated transcriptomic-metabolomic analysis reveals that cinnamaldehyde exposure positively regulates the phenylpropanoid pathway in postharvest Satsuma mandarin (Citrus unshiu). Pestic. Biochem. Physiol. 189, 105312. 10.1016/j.pestbp.2022.105312 36549824

[B27] Duan Y. Wang X. Jiao Y. Liu Y. Li Y. Song Y. (2024). Elucidating the role of exogenous melatonin in mitigating alkaline stress in soybeans across different growth stages: a transcriptomic and metabolomic approach. BMC Plant Biol. 24, 380. 10.1186/s12870-024-05101-9 38720246 PMC11077714

[B28] Evans N. Cloward J. Ward R. E. van Wietmarschen H. A. van Eekeren N. Kronberg S. L. (2024). Pasture-finishing of cattle in Western U.S. rangelands improves markers of animal metabolic health and nutritional compounds in beef. Sci. Rep. 14, 20240. 10.1038/s41598-024-71073-3 39215122 PMC11364752

[B29] FAO (2021). “Agriculture food systems transformation: from strategy to action,” in Forty-second session of the FAO conference, (Food and Agriculture Organization of the United Nations). Available online at: https://www.fao.org/family-farming/detail/en/c/1412070/.

[B30] Fayek N. M. Farag M. A. Saber F. R. (2021). Metabolome classification via GC/MS and UHPLC/MS of olive fruit varieties grown in Egypt reveal pickling process impact on their composition. Food Chem. 339, 127861. 10.1016/j.foodchem.2020.127861 32836025

[B31] Fayek N. M. Baky M. H. Li Z. Khalifa I. Capanoglu E. Farag M. A. (2024). Metabolome classification of olive by-products from different oil presses providing insights into its potential health benefits and valorization as analyzed via multiplex MS-based techniques coupled to chemometrics. Phytochem. Anal. 36, 2280–2300. 10.1002/pca.3385 38768954

[B32] Ferrari V. Gil G. Heinzen H. Zoppolo R. Ibáñez F. (2022). Influence of cultivar on nutritional composition and nutraceutical potential of Pecan growing in Uruguay. Front. Nutr. 9, 868054. 10.3389/fnut.2022.868054 35811969 PMC9257632

[B33] Fiehn O. (2002). Metabolomics – the link between genotypes and phenotypes. Plant Mol. Biol. 48, 155–171. 10.1023/A:1013713905833 11860207

[B34] Fu S. Wang L. Li C. Zhao Y. Zhang N. Yan L. (2024). Integrated transcriptomic, proteomic, and metabolomic analyses revealed molecular mechanism for salt resistance in soybean (Glycine max L.) seedlings. Int. J. Mol. Sci. 25, 13559. 10.3390/ijms252413559 39769326 PMC11678865

[B35] Gaikwad P. N. Sidhu G. S. Brar N. S. Singh J. Tokala V. Y. Sharma A. (2025). Roles of metabolites in fruit maturation, HLB-defense regulation and crosstalk between phytohormone signalling pathways in citrus. Plant Growth Regul. 105, 619–653. 10.1007/s10725-025-01308-4

[B36] Garcia-Aloy M. Groff N. Masuero D. Nisi M. Franco A. Battelini F. (2020). Exploratory analysis of commercial olive-based dietary supplements using untargeted and targeted metabolomics. Metabolites 10, 1–26. 10.3390/metabo10120516 33352793 PMC7766617

[B37] Ghisoni S. Lucini L. Angilletta F. Rocchetti G. Farinelli D. Tombesi S. (2019). Discrimination of extra-virgin-olive oils from different cultivars and geographical origins by untargeted metabolomics. Food Res. Int. 121, 746–753. 10.1016/j.foodres.2018.12.052 31108805

[B38] Giménez-Campillo C. Arroyo-Manzanares N. Campillo N. Díaz-García M. C. Viñas P. (2025). A volatilomic approach using ion mobility and mass spectrometry combined with multivariate chemometrics for the assessment of lemon juice quality. Food Control 169. 10.1016/j.foodcont.2024.111027

[B39] Goh R. M. V. Pua A. Luro F. Ee K. H. Huang Y. Marchi E. (2022). Distinguishing citrus varieties based on genetic and compositional analyses. PLoS One 17, e0267007. 10.1371/journal.pone.0267007 35436309 PMC9015143

[B40] Gómez J. F. M. Cônsolo N. R. B. Antonelo D. S. Beline M. Gagaoua M. Higuera-Padilla A. (2022). Impact of cattle feeding strategy on the beef metabolome. Metabolites 12, 640. 10.3390/metabo12070640 35888764 PMC9320084

[B41] Hamoud Y. A. AlGarawi A. M. Okla M. K. Sheteiwy M. S. Khalaf M. H. Alaraidh I. A. (2025). Metabolomic responses of wheat grains to olive mill wastewater and drought stress treatments. Sci. Rep. 15. 10.1038/s41598-025-98547-2 PMC12015521 40263511

[B42] He L. Hu Q. Zhang J. Xing R. Zhao Y. Yu N. (2023). An integrated untargeted metabolomic approach reveals the quality characteristics of black soybeans from different geographical origins in China. Food Res. Int. 169, 112908. 10.1016/j.foodres.2023.112908 37254343

[B43] Herrero M. Thornton P. K. Mason-D’Croz D. Palmer J. Benton T. G. Bodirsky B. L. (2020). Innovation can accelerate the transition towards a sustainable food system. Nat. Food 1, 266–272. 10.1038/s43016-020-0074-1

[B44] Hoffmann J. F. Carvalho I. R. Barbieri R. L. Rombaldi C. V. Chaves F. C. (2017a). Butia spp. (Arecaceae) LC-MS-based metabolomics for species and geographical origin discrimination. J. Agric. Food Chem. 65, 523–532. 10.1021/acs.jafc.6b03203 27984853

[B45] Hoffmann J. F. Zandoná G. P. dos Santos P. S. Dallmann C. M. Madruga F. B. Rombaldi C. V. (2017b). Stability of bioactive compounds in butiá (Butia odorata) fruit pulp and nectar. Food Chem. 237, 638–644. 10.1016/j.foodchem.2017.05.154 28764046

[B46] Imaz J. A. García S. González L. A. (2022). The metabolomics profile of growth rate in grazing beef cattle. Sci. Rep. 12, 2554. 10.1038/s41598-022-06592-y 35169253 PMC8847617

[B47] INIA (2024). Quiénes somos. Available online at: https://www.inia.uy/index.php/quienes-somos (Accessed July 6, 2025).

[B48] Islam N. Krishnan H. B. Slovin J. Natarajan S. (2023). Metabolic profiling of a fast neutron soybean mutant reveals an increased abundance of isoflavones. J. Agric. Food Chem. 71, 9994–10003. 10.1021/acs.jafc.3c01493 37343237

[B49] Jungen M. Gilcher C. Patz C. Steingass C. B. Schweiggert R. (2025). Phenolic compounds and quantitative 1H-NMR spectroscopy for authentication of lemon, lime, orange, and grapefruit juices. Food Chem. 485, 144379. 10.1016/j.foodchem.2025.144379 40288342

[B50] Kim S. S. Kim H. J. Park K. J. Kang S. B. Park Y. Han S. G. (2022). Metabolomic profiling of citrus unshiu during different stages of fruit development. Plants 11, 967. 10.3390/plants11070967 35406947 PMC9002680

[B51] King D. A. Shackelford S. D. Broeckling C. D. Prenni J. E. Belk K. E. Wheeler T. L. (2019). Metabolomic investigation of tenderness and aging response in beef longissimus steaks. Meat Muscle Biol. 3, 76–89. 10.22175/mmb2018.09.0027

[B52] Li J. Wang Y. Mukiibi R. Karisa B. Plastow G. S. Li C. (2022a). Integrative analyses of genomic and metabolomic data reveal genetic mechanisms associated with carcass merit traits in beef cattle. Sci. Rep. 12, 3389. 10.1038/s41598-022-06567-z 35232965 PMC8888742

[B53] Li M. Zhou J. Lang X. Han D. Hu Y. Ding Y. (2022b). Integrating transcriptomic and metabolomic analysis in roots of wild soybean seedlings in response to low-phosphorus stress. Front. Plant Sci. 13, 1006806. 10.3389/fpls.2022.1006806 36466240 PMC9713585

[B54] Li J. Wang Y. Z. Gmitter F. G. Wang Y. (2024). Identifying the earliest citrus responses to Candidatus Liberibacter asiaticus infection: a temporal metabolomics study. Front. Plant Sci. 15, 1455344. 10.3389/fpls.2024.1455344 39574442 PMC11579704

[B55] Liang X. Wang Y. Shen W. Liao B. Liu X. Yang Z. (2024). Genomic and metabolomic insights into the selection and differentiation of bioactive compounds in citrus. Mol. Plant 17, 1753–1772. 10.1016/j.molp.2024.10.009 39444162

[B56] Liebal U. W. Phan A. N. T. Sudhakar M. Raman K. Blank L. M. (2020). Machine learning applications for mass spectrometry-based metabolomics. Metabolites 10, 1–23. 10.3390/metabo10060243 32545768 PMC7345470

[B57] Liu H. M. Long C. R. Wang S. H. Fu X. M. Zhou X. Y. Mao J. M. (2021a). Transcriptome and metabolome comparison of smooth and rough citrus limon L. Peels grown on same trees and harvested in different seasons. Front. Plant Sci. 12, 749803. 10.3389/fpls.2021.749803 34691126 PMC8531254

[B58] Liu Y. Zhao F. Zhang Z. Li T. Zhang H. Xu J. (2021b). Structural diversity and distribution of limonoids in pummelo (Citrus grandis) fruit revealed by comprehensive UHPLC-MS/MS analysis. Sci. Hortic. 282, 109996. 10.1016/j.scienta.2021.109996

[B59] Liu X. Wang Z. Gmitter F. G. Grosser J. W. Wang Y. (2023). Effects of different rootstocks on the metabolites of huanglongbing-affected sweet Orange juices using a novel combined strategy of untargeted metabolomics and machine learning. J. Agric. Food Chem. 71, 1246–1257. 10.1021/acs.jafc.2c07456 36606748

[B60] Liu C. Zhang J. Liu H. Liu N. Sun L. Tan J. (2025a). Metabolic pathway analysis and potential biomarker identification of beef spoilage based on untargeted metabolomics. J. Sci. Food Agric. 106, 365–375. 10.1002/jsfa.70187 40934338

[B61] Liu H. Long C. Fu X. Wang S. Lou Y. Dong J. (2025b). Comparative global metabolome profile and transcriptome sequence analysis of the rough and smooth peel of the Orah Mandarin (Citrus reticulata). Horticulturae 11, 496. 10.3390/horticulturae11050496

[B62] Liu Q. Yu X. Jia F. Wen R. Sun C. Yu Q. (2025c). Comprehensive analyses of meat quality and metabolome alterations with aging under different aging methods in beef. Food Chem. 472, 142936. 10.1016/j.foodchem.2025.142936 39827567

[B63] López-Salas L. Díaz-Moreno J. Ciulu M. Borrás-Linares I. Quirantes-Piné R. Lozano-Sánchez J. (2024). Monitoring the phenolic and terpenic profile of olives, olive oils and By-Products throughout the production process. Foods 13. 10.3390/foods13101555 38790855 PMC11121151

[B64] Luzardo S. Banchero G. Ferrari V. Ibáñez F. Roig G. Aznárez V. (2021). Effect of fresh citrus pulp supplementation on animal performance and meat quality of feedlot steers. Animals 11, 3338. 10.3390/ani11123338 34944115 PMC8698122

[B65] Lyu W. Yuan B. Liu S. Simon J. E. Wu Q. (2022a). Assessment of lemon juice adulteration by targeted screening using LC-UV-MS and untargeted screening using UHPLC-QTOF/MS with machine learning. Food Chem. 373, 131424. 10.1016/j.foodchem.2021.131424 34710685

[B66] Lyu X. Sun C. Zhang J. Wang C. Zhao S. Ma C. (2022b). Integrated proteomics and metabolomics analysis of nitrogen system regulation on soybean plant nodulation and nitrogen fixation. Int. J. Mol. Sci. 23, 2545. 10.3390/ijms23052545 35269687 PMC8910638

[B67] Maciá-Vázquez A. A. Núñez-Gómez D. Martínez-Nicolás J. J. Legua P. Melgarejo P. (2023). Morphological and biochemical characterization of late-season varieties of mandarin growing in Spain under homogeneous growing conditions. Agronomy 13. 10.3390/agronomy13071825

[B68] Mani V. Park S. Lee K. Kim J. A. Ha K. Park S. K. (2024). Metabolic perspective on soybean and its potential impacts on digital breeding: an updated overview. J. Plant Biol. 67, 87–98. 10.1007/s12374-023-09419-z

[B69] Migues I. Hodos N. Moltini A. I. Gámbaro A. Rivas F. Moyna G. (2021). 1H NMR metabolic profiles as selection tools of new mandarin cultivars based on fruit acceptability. Sci. Hortic. 287, 110262. 10.1016/j.scienta.2021.110262

[B70] Migues I. Rivas F. Moyna G. Kelly S. D. Heinzen H. (2022). Predicting mandarin fruit acceptability: from high-field to benchtop NMR spectroscopy. Foods. 10.3390/foods11150000 PMC9407331 36010384

[B71] Misra B. B. Langefeld C. Olivier M. Cox L. A. (2019). Integrated omics: tools, advances and future approaches. J. Mol. Endocrinol. 62, R21–R45. 10.1530/JME-18-0055 30006342

[B72] Mokhtari M. Jackson M. D. Brown A. S. Ackerley D. F. Ritson N. J. Keyzers R. A. (2018). Bioactivity-guided metabolite profiling of feijoa (Acca sellowiana) cultivars identifies 4-Cyclopentene-1,3-dione as a potent antifungal inhibitor of chitin synthesis. J. Agric. Food Chem. 66, 5531–5539. 10.1021/acs.jafc.7b06154 29546758

[B73] Monribot-Villanueva J. L. Altúzar-Molina A. Aluja M. Zamora-Briseño J. A. Elizalde-Contreras J. M. Bautista-Valle M. V. (2022). Integrating proteomics and metabolomics approaches to elucidate the ripening process in white Psidium guajava. Food Chem. 367. 10.1016/j.foodchem.2021.130656 34359004

[B74] Montoro P. Serreli G. Gil K. A. D’Urso G. Kowalczyk A. Tuberoso C. I. G. (2020). Evaluation of bioactive compounds and antioxidant capacity of edible feijoa (Acca sellowiana (O. Berg) Burret) flower extracts. J. Food Sci. Technol. 57, 2051–2060. 10.1007/s13197-020-04239-2 32431331 PMC7230090

[B75] Nezamivand-Chegini M. Metzger S. Moghadam A. Tahmasebi A. Koprivova A. Eshghi S. (2023). Integration of transcriptomic and metabolomic analyses provides insights into response mechanisms to nitrogen and phosphorus deficiencies in soybean. Plant Sci. 326, 111498. 10.1016/j.plantsci.2022.111498 36252857

[B76] Nguyen K. O. T. Do T. N. Dang K. P. Sato M. Hirai M. Y. (2024). Single-grain-based widely targeted metabolomics profiling of sixty-four accessions of Japanese wild soybean (Glycin soja Sieb. Et Zucc.). Int. J. Food Sci. Technol. 59, 4251–4262. 10.1111/ijfs.16654

[B77] Padhi E. M. T. Maharaj N. Lin S. Y. Mishchuk D. O. Chin E. Godfrey K. (2019). Metabolome and microbiome signatures in the roots of citrus affected by huanglongbing. Phytopathology 109, 2022–2032. 10.1094/PHYTO-03-19-0103-R 31433274

[B78] Parri S. Cai G. Romi M. Cantini C. Pinto D. C. G. A. Silva A. M. S. (2024). Comparative metabolomics of leaves and stems of three Italian olive cultivars under drought stress. Front. Plant Sci. 15, 1408731. 10.3389/fpls.2024.1408731 39022609 PMC11251969

[B79] Pascoal G. B. Meza S. L. R. Tobaruela E. C. Franzon R. C. Massaretto I. L. Purgatto E. (2025). Volatile and non-volatile compounds profiling of Brazilian pitanga (Eugenia uniflora L.) varieties during ripening using gas chromatography-mass spectrometry approach. J. Braz Chem. Soc. 36. 10.21577/0103-5053.20240196

[B80] Pedrini C. A. Machado F. S. Fernandes A. R. M. Cônsolo N. R. B. Ocampos F. M. M. Colnago L. A. (2024). Performance, meat quality and meat metabolomics outcomes: efficacy of 3-Nitrooxypropanol in feedlot beef cattle diets. Animals 14, 2576. 10.3390/ani14172576 39272361 PMC11394267

[B81] Pereira M. Tissot F. Faccio R. Ibáñez F. Pistón M. (2021). A simple and economical ultrasound-assisted method for Cd and Pb extraction from fruits and vegetables for food safety assurance. Results Chem. 3, 100089. 10.1016/j.rechem.2020.100089

[B82] Phoemchalard C. Uriyapongson S. Tathong T. Pornanek P. (2022). 1H NMR metabolic profiling and meat quality in three beef cattle breeds from Northeastern Thailand. Foods 11, 3821. 10.3390/foods11233821 36496627 PMC9736620

[B83] Polizel G. H. G. Diniz W. J. S. Cesar A. S. M. Ramírez-Zamudio G. D. Cánovas A. Dias E. F. F. (2025). Impacts of prenatal nutrition on metabolic pathways in beef cattle: an integrative approach using metabolomics and metagenomics. BMC Genomics 26, 359. 10.1186/s12864-025-11545-6 40211121 PMC11983759

[B84] Quintela A. L. Santos M. F. C. de Lima R. F. Mayer J. L. S. Marcheafave G. G. Arruda M. A. Z. (2024). Influence of silver nanoparticles on the metabolites of two transgenic soybean varieties: an NMR-based metabolomics approach. J. Agric. Food Chem. 72, 12281–12294. 10.1021/acs.jafc.4c00756 38747520 PMC11140748

[B85] Raikar S. V. Isak I. Patel S. Newson H. L. Hill S. J. (2023). Establishment of feijoa (Acca sellowiana) callus and cell suspension cultures and identification of arctigenin - a high value bioactive compound. Front. Plant Sci. 14, 1281733. 10.3389/fpls.2023.1281733 38298607 PMC10829094

[B86] Rao G. Zhang J. Liu X. Li X. Wang C. (2021). Combined metabolome and transcriptome profiling reveal optimal harvest strategy model based on different production purposes in olive. Foods 10, 360. 10.3390/foods10020360 33562421 PMC7915097

[B87] Razzaq A. Wishart D. S. Wani S. H. Hameed M. K. Mubin M. Saleem F. (2022). Advances in metabolomics-driven diagnostic breeding and crop improvement. Metabolites 12, 511. 10.3390/metabo12060511 35736444 PMC9228725

[B88] Ren S. Hinzman A. A. Kang E. L. Szczesniak R. D. Lu L. J. (2015). Computational and statistical analysis of metabolomics data. Metabolomics 11, 1492–1513. 10.1007/s11306-015-0823-6

[B89] Rha C. S. Jang E. K. Lee J. S. Kim J. S. Ko M. J. Lim S. (2023). Statistical discrimination using different machine learning models reveals dissimilar key compounds of soybean leaves in targeted polyphenol-metric metabolomics in terms of traits and cultivation. Food Chem. 404, 134454. 10.1016/j.foodchem.2022.134454 36240552

[B90] Ros A. D. Masuero D. Riccadonna S. Bubola K. B. Mulinacci N. Mattivi F. (2019). Complementary untargeted and targeted metabolomics for differentiation of extra virgin olive oils of different origin of purchase based on volatile and phenolic composition and sensory quality. Molecules 24. 10.3390/molecules24162896 PMC6720806 31404955

[B91] Schalch Junior F. J. Polizel G. H. G. Cançado F. A. C. Q. Fernandes A. C. Mortari I. Pires P. R. L. (2022). Prenatal supplementation in beef cattle and its effects on plasma metabolome of dams and calves. Metabolites 12, 347. 10.3390/metabo12040347 35448533 PMC9028846

[B92] Schripsema J. (2019). Similarity and differential NMR spectroscopy in metabolomics: application to the analysis of vegetable oils with 1H and 13C NMR. Metabolomics 15, 39. 10.1007/s11306-019-1502-9 30843128

[B93] Senizza B. Ganugi P. Trevisan M. Lucini L. (2023). Combining untargeted profiling of phenolics and sterols, supervised multivariate class modelling and artificial neural networks for the origin and authenticity of extra-virgin olive oil: a case study on Taggiasca Ligure. Food Chem. 404, 134543. 10.1016/j.foodchem.2022.134543 36240558

[B94] Serrano-García I. Martakos I. C. Olmo-García L. León L. de la Rosa R. Gómez-Caravaca A. M. (2024). Application of liquid chromatography-ion mobility spectrometry-mass spectrometry-based metabolomics to investigate the basal chemical profile of olive cultivars differing in verticillium dahliae resistance. J. Agric. Food Chem. 10.1021/acs.jafc.4c07155 39578263 PMC11638956

[B95] Song X. Dai F. Yao J. Li Z. Huang Z. Liu H. (2023). Characterization of the volatile profile of feijoa (Acca sellowiana) fruit at different ripening stages by HS-SPME-GC/MS. LWT 184, 115011. 10.1016/j.lwt.2023.115011

[B96] Spears M. Cooper G. Sather B. Bailey M. Boles J. A. Bothner B. (2024). Comparative impact of organic grass-fed and conventional cattle-feeding systems on beef and human postprandial metabolomics—A randomized clinical trial. Metabolites 14, 533. 10.3390/metabo14100533 39452914 PMC11509860

[B97] Suh J. H. Tang X. Zhang Y. Gmitter F. G. Wang Y. (2021). Metabolomic analysis provides new insight into tolerance of huanglongbing in citrus. Front. Plant Sci. 12, 710598. 10.3389/fpls.2021.710598 34421957 PMC8371912

[B98] Sun F. Piao M. Zhang X. Zhang S. Wei Z. Liu L. (2022). Multi-omics analysis of transcriptomic and metabolomics profiles reveal the molecular regulatory network of marbling in early castrated holstein steers. Animals 12, 3398. 10.3390/ani12233398 36496924 PMC9736081

[B99] Sun D. Mu B. Liu Y. Zhao C. Li H. Wang J. (2024). Widely targeted metabolomic analysis reveals dynamic metabolic changes in yanbian cattle during dry-aging process. Foods 13, 2879. 10.3390/foods13182879 39335808 PMC11430874

[B100] Taheri S. de Andrade J. C. Conte-Junior C. A. (2024). Emerging perspectives on analytical techniques and machine learning for food metabolomics in the era of industry 4.0: a systematic review. Crit. Rev. Food Sci. Nutr. 65, 6045–6071. 10.1080/10408398.2024.2435597 39621023

[B101] Takpah D. Asghar M. A. Raza A. Javed H. H. Ullah A. Huang X. (2023). Metabolomics analysis reveals soybean node position influence on metabolic profile of soybean seed at various developmental stages. J. Plant Growth Regul. 42, 6788–6800. 10.1007/s00344-023-11048-2

[B102] Tang N. Chen N. Hu N. Deng W. Chen Z. Li Z. (2018). Comparative metabolomics and transcriptomic profiling reveal the mechanism of fruit quality deterioration and the resistance of citrus fruit against Penicillium digitatum. Postharvest Biol. Technol. 145, 61–73. 10.1016/j.postharvbio.2018.06.007

[B103] Tang F. Polari J. J. Green H. S. Wang S. C. Hatzakis E. (2022). NMR-based metabolomics for olive oil cultivar classification: a comparison with standard targeted analysis of fatty acids and triglycerides. Food Control 137, 108939. 10.1016/j.foodcont.2022.108939

[B104] Tareq F. S. Kotha R. R. Natarajan S. Sun J. Luthria D. L. (2023). An untargeted metabolomics approach to study the variation between wild and cultivated soybeans. Molecules 28, 5507. 10.3390/molecules28145507 37513379 PMC10386028

[B105] Tenório C. J. L. dos Santos Dantas T. Abreu L. S. Ferreira M. R. A. Soares L. A. L. (2024). Influence of major polyphenols on the anti-candida activity of Eugenia uniflora leaves: isolation, LC-ESI-HRMS/MS characterization and *in vitro* evaluation. Molecules 29, 2761. 10.3390/molecules29122761 38930827 PMC11206001

[B106] Tian H. Lam S. M. Shui G. (2016). Metabolomics,a powerful tool for agricultural research. Int. J. Mol. Sci. 17. 10.3390/ijms17111871 27869667 PMC5133871

[B107] Tian R. Kharrati-Koopaee H. Asadollahpour Nanaie H. Wang X. Zhao M. Li H. (2023). Comparative metabolomics analysis shows key metabolites as potential biomarkers for selection of beef fat colour. Anim. Prod. Sci. 63, 1063–1067. 10.1071/an22476

[B108] Traband R. C. Wang X. Resendiz M. Meng M. Hiraoka Y. Jia Q. (2025). A novel approach for comparing selected metabolites in citrus leaves and fruits across datasets. Plants 14, 1406. 10.3390/plants14101406 40430972 PMC12115042

[B109] Uruguay XXI (2024a). Informe del Sector Agrícola - 2024. Available online at: https://www.uruguayxxi.gub.uy/es/centro-informacion/articulo/informe-agricola-2024/ (Accessed July 10, 2025).

[B110] Uruguay XXI (2024b). Informe sector Ganadero - 2024. Available online at: https://www.uruguayxxi.gub.uy/es/centro-informacion/articulo/informe-sector-ganadero-2024/ (Accessed July 10, 2025).

[B111] Valdés A. Álvarez-Rivera G. Socas-Rodríguez B. Herrero M. Ibáñez E. Cifuentes A. (2022). Foodomics: analytical opportunities and challenges. Anal. Chem. 94, 366–381. 10.1021/acs.analchem.1c04678 34813295 PMC8756396

[B112] Verpoorte R. Choi Y. H. Kim H. K. (2005). Ethnopharmacology and systems biology: a perfect holistic match. J. Ethnopharmacol. 100, 53–56. 10.1016/j.jep.2005.05.033 16026949

[B113] Verpoorte R. Choi Y. H. Mustafa N. R. Kim H. K. (2008). Metabolomics: back to basics. Phytochem. Rev. 7, 525–537. 10.1007/s11101-008-9091-7

[B114] Vital R. G. Müller C. Freire F. B. S. Silva F. B. Batista P. F. Fuentes D. (2022). Metabolic, physiological and anatomical responses of soybean plants under water deficit and high temperature condition. Sci. Rep. 12, 16467. 10.1038/s41598-022-21035-4 36183028 PMC9526742

[B115] Wang F. Huang Y. Wu W. Zhu C. Zhang R. Chen J. (2020). Metabolomics analysis of the peels of different colored citrus fruits (Citrus reticulata cv. ‘Shatangju’) during the maturation period based on UHPLC-QQQ-MS. Molecules 25. 10.3390/molecules25020396 31963595 PMC7024170

[B116] Wang Z. Gmitter F. G. Grosser J. W. Wang Y. (2022a). Natural sweeteners and sweetness-enhancing compounds identified in citrus using an efficient metabolomics-based screening strategy. J. Agric. Food Chem. 70, 10593–10603. 10.1021/acs.jafc.2c03515 35980814

[B117] Wang Z. Niu Y. Vashisth T. Li J. Madden R. Livingston T. S. (2022b). Nontargeted metabolomics-based multiple machine learning modeling boosts early accurate detection for citrus Huanglongbing. Hortic. Res. 9, uhac145. 10.1093/hr/uhac145 36061619 PMC9433982

[B118] Wang J. Zheng Q. Wang C. Zhou A. (2024a). Classification of soybeans from different habitats based on metabolomic–transcriptomic integration. Appl. Biol. Chem. 67, 30. 10.1186/s13765-024-00882-x

[B119] Wang M. Chen Y. Li S. Yu J. Yang L. Hong L. (2024b). Widely targeted metabolomic analysis provides new insights into the effect of rootstocks on citrus fruit quality. Metabolites 14, 242. 10.3390/metabo14040242 38668370 PMC11052146

[B120] Wang S. Shen S. Wang C. Wang X. Yang C. Zhou S. (2024c). A metabolomics study in citrus provides insight into bioactive phenylpropanoid metabolism. Hortic. Res. 11, uhad267. 10.1093/hr/uhad267 38304332 PMC10831325

[B121] Wang Y. Liu W. Li W. Wang C. Dai H. Xu R. (2024d). Integrative analysis of metabolome and transcriptome reveals regulatory mechanisms of flavonoid biosynthesis in soybean under salt stress. Front. Plant Sci. 15, 1415867. 10.3389/fpls.2024.1415867 38957602 PMC11217524

[B122] Wu W. Zhang L. Zheng X. Huang Q. Farag M. A. Zhu R. (2022). Emerging applications of metabolomics in food science and future trends. Food Chem. X 16, 100500. 10.1016/j.fochx.2022.100500 36519105 PMC9743159

[B123] Xiao P. Qu J. Wang Y. Fang T. Xiao W. Wang Y. (2024). Transcriptome and metabolome atlas reveals contributions of sphingosine and chlorogenic acid to cold tolerance in Citrus. Plant Physiol. 196, 634–650. 10.1093/plphys/kiae327 38875157

[B135] Xu C. Chen Q. Xu Y. He Y. Hong D. Xiao H. (2025). Transcriptomics and Metabolomics Integrated Analysis Provide Insights Into the Differential Accumulation of Bitterness in Pummelo (Citrus grandis). Physiol. Plant. 177 (4), e70374. 10.1111/ppl.70374 40628692

[B124] Xu L. Xu Z. Kelly S. Liao X. (2020). Integrating untargeted metabolomics and targeted analysis for not from concentrate and from concentrate orange juices discrimination and authentication. Food Chem. 329, 127130. 10.1016/j.foodchem.2020.127130 32516707

[B125] Xu W. Wang Q. Zhang W. Zhang H. Liu X. Song Q. (2022). Using transcriptomic and metabolomic data to investigate the molecular mechanisms that determine protein and oil contents during seed development in soybean. Front. Plant Sci. 13, 1012394. 10.3389/fpls.2022.1012394 36247601 PMC9557928

[B126] Xu W. Li M. Li W. Liu H. Xu X. Yang T. (2024). Effect of H2 treatment under UV-B irradiation on the enrichment of germinated soybean isoflavones and mechanisms based on growth state, antioxidant system, and metabolomics. LWT 195, 115821. 10.1016/j.lwt.2024.115821

[B127] Zahran H. A. Fayez S. Zayed A. Azab M. A. Fayek N. M. Zhang L. (2025). Integrated metabolomic profiling of olive oil, olive mill wastewater, and pomace from Egyptian cultivars based on UHPLC-MS/MS and NMR coupled with chemometrics. J. Adv. Res. 10.1016/j.jare.2025.08.068 40902894 PMC12869296

[B128] Zeng J. Chen C. Chen M. Chen J. (2022). Comparative transcriptomic and metabolomic analyses reveal the delaying effect of naringin on postharvest decay in citrus fruit. Front. Plant Sci. 13, 1045857. 10.3389/fpls.2022.1045857 36531365 PMC9748555

[B129] Zhang J. Sun M. Elmaidomy A. H. Youssif K. A. Zaki A. M. M. Kamal H. H. (2023a). Emerging trends and applications of metabolomics in food science and nutrition. Food Funct. 14, 9050–9082. 10.1039/d3fo01770b 37740352

[B130] Zhang W. Hou H. Zhang D. Zhu B. Yuan H. Gao T. (2023b). Transcriptomic and metabolomic analysis of soybean nodule number improvements with the use of water-soluble humic materials. J. Agric. Food Chem. 71, 197–210. 10.1021/acs.jafc.2c06200 36573896

[B131] Zhang L. Iannotti F. A. Saber F. R. Arafa R. K. Moriello A. S. Rasle R. A. (2025a). The phenolic signature of Psidium cattleianum fruits and leaves modulates TRPV1 and TRPA1 transient receptor potential channels: a metabolomics, *in vitro,* and *in silico* study. Food Sci. Nutr. 13. 10.1002/fsn3.70075 PMC11931593 40129993

[B132] Zhang X. Liu J. Zhang R. Li J. Li J. Zhang D. (2025b). Transcriptomic and metabolomic explanation of the interaction between soybean and root rot caused by Fusarium tricinctum. J. Agric. Food Chem. 73, 19944–19957. 10.1021/acs.jafc.5c02223 40734228

[B133] Zhu D. Guan D. Fan B. Sun Y. Wang F. (2022a). Untargeted mass spectrometry-based metabolomics approach unveils molecular changes in heat-damaged and normal soybean. LWT 171, 114136. 10.1016/j.lwt.2022.114136

[B134] Zhu L. Yang Q. Yu X. Fu X. Jin H. Yuan F. (2022b). Transcriptomic and metabolomic analyses reveal a potential mechanism to improve soybean resistance to anthracnose. Front. Plant Sci. 13, 850829. 10.3389/fpls.2022.850829 35574068 PMC9094087

